# Static plantar pressure and functional capacity in children with femoral shaft fractures treated by titanium elastic nailing

**DOI:** 10.1186/s12891-019-2951-z

**Published:** 2019-11-26

**Authors:** Elena Amăricăi, Oana Suciu, Roxana Ramona Onofrei, Emil Radu Iacob, Daniela Iacob, Călin Marius Popoiu, Marius Negru, Oana Belei, Luminița Bădițoiu, Eugen Boia

**Affiliations:** 1 Department of Rehabilitation, Physical Medicine and Rheumatology, “Victor Babes” University of Medicine and Pharmacy, “Louis Țurcanu” Emergency Children’s Hospital, Timisoara, Romania; 2 Department of Rehabilitation, Physical Medicine and Rheumatology, “Victor Babes” University of Medicine and Pharmacy, “Pius Brînzeu” Emergency County Hospital, Timisoara, Romania; 3 Department of Paediatric Surgery, “Victor Babes” University of Medicine and Pharmacy, “Louis Țurcanu” Emergency Children’s Hospital, Timisoara, Romania; 4 Department of Neonatology, “Victor Babes” University of Medicine and Pharmacy, “Pius Brînzeu” Emergency County Hospital, Timisoara, Romania; 5 “Louis Țurcanu” Emergency Children’s Hospital, Timisoara, Romania; 6 1st Pediatric Department, “Victor Babes” University of Medicine and Pharmacy, “Louis Țurcanu” Emergency Children’s Hospital, Timisoara, Romania; 7 Epidemiology Department, “Victor Babes” University of Medicine and Pharmacy, Timisoara, Romania

**Keywords:** Femoral shaft fracture, Children, Plantar pressure, Barycentre, Functional capacity

## Abstract

**Background:**

Femoral shaft fractures represent common fractures of the lower limb in the paediatric population. The objectives of our study were to analyse the static plantar pressure and functional capacity in children with surgically treated unilateral femoral shaft fractures, 1 month after the metallic implant removal.

**Methods:**

Our study included 24 children with unilateral femoral shaft fracture (fracture site contralateral to the dominant leg) treated by titanium elastic nailing (TEN) implants, with the removal of the implant 6 months after initial surgery. The patients were divided into two groups: Group 1 (12 patients without inpatient rehabilitation) and Group 2 (12 patients who attended inpatient rehabilitation). The patients and 12 gender and age-matched healthy controls performed plantar pressure analysis and functional capacity testing (6-min walk test: 6MWT). For patients in Group 1 and 2 the assessments were performed 1 month after the TEN implant removal. Paired t-tests were used to compare the intragroup data. A one-way ANOVA test for independent measures was performed to assess the differences for plantar pressure, and 6MWT among study groups and controls.

**Results:**

All study patients had left femoral shaft fractures (affected limb). The patients and controls were all right leg dominant. In both Group 1 and Group 2 total foot loading was significantly higher on the non-affected limb compared with the affected limb. When compared to the non-affected limb, the loadings on the affected limb were significantly increased on the first and fifth metatarsal in Group 1, and on the fifth metatarsal in Group 2, with a significantly smaller heel loading. When compared with the controls we found significant differences for all pressure parameters, except for the right foot load for the rehabilitation group. Although the functional capacity values were higher in the rehabilitation group the two patients groups had significantly lower 6MWT values.

**Conclusions:**

When compared to healthy controls children with surgically treated unilateral femoral shaft fractures, although assessed after 7 months, had a different weight distribution on the feet and a decreased functional capacity. A rehabilitation programme included in the management of these patients is important for regaining their functional level.

## Introduction

Fractures of the femoral shaft represent common fractures of the lower limb in the paediatric population [1]. Although constituting less than 2% of all fractures in children they are an important burden on healthcare systems and families as they are among the most frequent causes requiring hospitalization for paediatric orthopaedic injuries [2]. The studies reported an annual incidence of femoral shaft fractures between 9.5–13.3 in girls and 21.9–37 in boys per 100,000 children [3–6].

The majority of the paediatric femoral shaft fractures are now treated operatively, with economic and social benefits over conservative treatment [7]. Surgical treatment by using titanium elastic nailing (TEN) proved its efficacy when referring to hospitalization period, duration until assistive or independent walking, and returning to school [8]. However, after a lower limb fracture the loading of the musculoskeletal system is affected, influencing thus the support and balance control of the body [9, 10].

Plantar pressure measurement during bipedal standing provides important information of foot loading under various postural static activities [11], as well as information related to human gait and posture evaluation [12]. It can be determined by static systems consisting of easy-to-use baropodometric devices that involve pressure mats [13].

The 6-min walk test (6MWT) represents a way of assessing the functional capacity. It is a submaximal test that offers a measure for responses of cardiopulmonary and musculoskeletal systems involved in exercise [14].

Our rationale to perform the current study was to take into consideration children with unilateral femoral shaft fractures who followed surgical treatment by TEN implants. This category of paediatric patients represents a group not very accurately assessed in contrast tothe adult patients with different types of lower limb fractures. The hypothesis of the current work was that there would be differences in static plantar pressure parameters and 6MWT between the children with operatively treated unilateral femoral shaft fractures and healthy controls, with better results for those patients who also followed a rehabilitation programme.

The objectives of our study were to analyse the static plantar pressure and functional capacity in children with surgically treated unilateral femoral shaft fractures 1 month after the removal of the metallic implant.

## Material and methods

### Participants

The parents of 29 children were asked to participate in the study. The children were selected from the patients addressed to the Paediatric Surgery, “Louis Turcanu” Emergency Children’s Hospital Timisoara, Romania. Inclusion criteria were: unilateral femoral shaft fracture (transverse or short oblique type, stable pattern) surgically treated by TEN implants, fracture site contralateral to the dominant leg. The dominant leg was considered to be the self-reported, preferred kicking leg [15].

Exclusion criteria were: pathological femoral fractures, additional injury apart from the femoral fracture, other musculoskeletal disorders, history of neurological diseases, vestibular or visual disturbances or any other pathology that would impair their motor performance, age younger than 5 years. Five children met exclusion criteria: femoral fracture secondary to osteoid osteoma (1), tibial shaft fracture (1), metatarsal fracture (1), cerebral palsy (1) and age of 4 years (1).

We have enrolled 24 patients. The patients were divided into two groups: 12 patients who did not attend inpatient rehabilitation (Group 1) and 12 patients who attended inpatient rehabilitation programme after the initial femoral fracture surgery (Group 2). Twelve gender and age-matched healthy controls were recruited by posters placed at public schools nearby. The sample size was calculated using the ClinCalc application [16]. For an estimated incidence of functioning in children with femoral shaft fractures of 50% and in healthy children of 99.99%, the minimum sample size for a power of 0.8, α of 0.05 was of 11 subjects per group.

Participation in the study was voluntary. Written informed consent was obtained from all the participants’ parents. The study has been carried out in accordance with the Declaration of Helsinki and was approved by Institutional Ethics Committee (“Louis Turcanu” Emergency Children’s Hospital, No 56/05.10.2018).

### Rehabilitation programme

In the first 4 weeks after surgery all patients were taught ankle pumps exercises, isometric straight leg raises, quadriceps and hamstrings strengthening exercises with active knee range of motion. Non weight-bearing walking with crutches was started 3 days after surgery. After the 4th postoperative week the rehabilitation group began a daily physical therapy programme for a 2-week period as inpatients in the Rehabilitation department. The mobility exercises progressed to stool sliding, supine wall sliding, stretching of the hamstrings, gastrocnemius and soleus muscles. Strengthening of quadriceps and hip abductors progressed from isometric exercises to toe raises, bilateral minisquats, and abduction activities with Thera-band. Proprioceptive activities including balance board activities, steps on mini-trampoline were initiated at the end of the 5th week. Weight bearing as tolerated was initiated with full weight bearing in week 6. After the inpatient rehabilitation, Group 2 patients followed a home exercise programme with increased mobility, strengthening, and proprioception exercises, and full-weight bearing.

### Assessment

The following patients’ and controls’ characteristics were collected: age, height, weight, body mass index, leg length. Leg length was measured in centimetres with children lying supine, from anterior superior iliac spine to the ipsilateral medial malleolus, with a standard tape measure, on each lower limb. We took into consideration the leg length discrepancy.

The protocol of our study consisted in one assessment for Groups 1 and 2, and controls. Each evaluation involved the static plantar pressure analysis and functional capacity testing (6MWT) both for the study groups and the controls. For patients in Group 1 and 2 the assessments were performed 1 month after the TEN implant removal. The removal of the implant was performed 6 months after the initial surgery.

### Plantar pressure analysis

PoData system (Chinesport, Udine, Italy) was used for plantar pressure assessment (Additional file 1). The system provides information about weight distribution, barycentre and stabilometry [17]. The children were asked to stand on the platform, barefoot, in upright posture, lower limbs extended and arms positioned naturally along their sides, eyes opened, for 20 s. The feet were positioned at an angle of 30° to each other and 5 cm between the heels. The children were instructed to look ahead, fixing a target point on the wall, not to talk or move. The testing was considered invalid and repeated if at least one of the following errors were observed: the child moved or lifted an arm or both, lifting the forefoot or the heel, falling out of position, moving the head or talking.

Plantar pressure was recorded on three anatomical regions: first and fifth metatarsal heads, and heel, in both right and left foot [18]. Percentage of body weight distribution was calculated for each area. An ideal load of an ideal subject has the following distribution: 1/6 (16.67%) of total weight on fifth metatarsal head, 2/6 (33.33%) of total weight on first metatarsal head, and 3/6 (50%) of total weight on heel [19].

The measured body centre of pressure was compared with the theoretical one [20, 21]. Body centre of pressure deviation from theoretical reference was measured on anterior-posterior axis (Y-axis) and latero-lateral axis (X-axis or left-right axis) [17, 20, 22]. Average distances from the ideal barycenter were provided by the software for both axes. A positive value represents an anterior deviation on the anterior-posterior axis, and a right deviation on the latero-lateral axis.

### Functional capacity assessment

The 6MWT was conducted according to a standardized protocol. The subject was instructed to walk up and down a measured corridor, covering as much ground as possible over a 6-min period. The wording of encouragement during the testing was standardized (“keep going”, “you are doing fine”, “everything is going well”) and given by the same person at set times during the test. The 6-min walk distance was also recorded. Pulse and oxygen saturation were recorded during the test [23].

### Statistical analysis

Statistical analysis was performed using MedCalc version 8.11 (MedCalc Software bvba, Ostend, Belgium) [24]. All data were tested for normality with the Shapiro-Wilk’s test. The homogeneity of variances between groups was tested with Levene’s test. Descriptive statistics were computed for all variables (mean and standard deviation). Paired t-tests were used to compare the intragroup data. A one-way ANOVA test for independent measures was performed to assess the differences for anthropometric data, plantar pressure, both for right and left foot, and 6MWT among Group 1, Group 2 and healthy controls. A Tukey-Kramer post-hoc test for all pair wise comparisons was also performed. The significance level was set at *p* < 0.05 for all tests (Additional file 2).

## Results

The two patients groups and the control group were homogenous in terms of anthropometrical characteristics (Levene’s test - *p* > 0.05) (Table 1).

**Table 1:** Patients and controls characteristics

Variables	Group 1 (*N* = 12)	Group 2 (*N* = 12)	Controls (*N* = 12)
Age (years)^a^	14.67 ± 2.18	14.58 ± 2.11	14.67 ± 2.18
Height (cm)^a^	163.25 ± 15.37	163.58 ± 15.87	163.67 ± 15.08
Weight (kg)^a^	54.67 ± 12.31	54.83 ± 12.52	55.67 ± 13.07
BMI (kg/m^2^)^a^	21.23 ± 1.57	20.22 ± 1.45	20.48 ± 1.86
Fracture type			
Transverse, N	6	6	
Short oblique, N	6	6	
Leg length discrepancy (cm)^a^	0.95 ± 0.37	0.97 ± 0.35	0.79 ± 0.32
Gender			
Female, N	3	3	3
Male, N	9	9	9

*N* Number of subjects, *BMI* Body mass index

^a^Data are presented as mean ± standard deviation

All patients in Group 1 and 2 had left femoral shaft fractures (affected limb). The patients and controls were all right leg dominant.

Results of the plantar pressure analysis and 6MWT are presented in Table 2. In both Group 1 and Group 2 total foot loading was significantly higher on the non-affected limb compared with the affected limb (*p* < 0.001). In Group 1 the loadings on first and fifth metatarsal were significantly increased on the left foot (affected limb) when compared with the right foot (non-affected limb) (*p* < 0.01). In contrast, the heel loading was significantly smaller on the left foot (affected limb) in comparison to the right one (non-affected limb) (*p* < 0.001). In Group 2 (rehabilitation group), when compared to the right foot, the left foot (affected site) had a significantly increased loading on fifth metatarsal (*p* = 0.001) and a significantly lower load on the heel (*p* < 0.0001). In healthy children no significant differences were noticed between limbs (*p* > 0.05) when considering the three sites of weight distribution (first and fifth metatarsal heads, and heel).

**Table 2:** Pressure load distribution (percentage of body weight on each foot) and functional capacity in patients and controls

Variables	Group 1 (*N* = 12)	*P*^#^	Group 2 (*N* = 12)	*P*^#^	Controls (*N* = 12)	*P*^#^
Right foot load(%)	56.25 ± 2.92 *	< 0.0001	53.75 ± 2.42	0.0001	51 ± 3.13	NS
Left foot load (%)	43.75 ± 2.92 *		45.75 ± 2.63 ‡		49 ± 3.13	
Right MT1 (%)	20.91 ± 2.15 *†	0.002	28.75 ± 3.28 ‡	NS	32.67 ± 2.49	NS
Left MT1 (%)	24 ± 3.04 *†		29.41 ± 3.78 ‡		32.75 ± 2.92	
Right MT5 (%)	39 ± 3.33 *†	< 0.0001	25.08 ± 4.64‡	0.001	17.16 ± 1.8	NS
Left MT5 (%)	52.25 ± 5.01 *†		32.92 ± 5.28 ‡		17 ± 2.13	
Right heel (%)	40.25 ± 3.81 *†	< 0.0001	46.41 ± 2.84 ‡	< 0.0001	50.33 ± 2.14	NS
Left heel (%)	22.91 ± 2.81 *†		37.67 ± 3.77 ‡		50.58 ± 2.15	
6MWT (meters)	412.5 ± 45.18 *		439.17 ± 41.76 ‡		505.5 ± 93.51	

*MT1* First metatarsal head, *MT5* Fifth metatarsal head, *6MWT* 6-min walk test, *NS* Not significant

* different from controls at *p* < 0.05; † different from Group 2 at *p* < 0.05;

‡ different from controls at *p* < 0.05; ^#^comparison between the right and left limb (paired t-test)

There were statistically significant differences among the compared groups (Groups 1 and 2, and controls) as determined by one-way ANOVA for all pressure parameters (with a large effect size - η^2^_p_ > 0.377), except for the right foot load (between rehabilitation group and controls, and between the two study groups) and left foot loading between the two patients groups.

Significant differences were also observed for the 6MWT between patients groups and controls (F_2,33_ = 6.59, *p* = 0.004, η^2^_p_ > 0.285). Post-hoc analysis revealed that although the functional capacity values were higher in the rehabilitation group, there were no significant differences between the two patients groups. When compared to healthy controls, the 6MWT was significantly lower in both Group 1 and Group 2.

In Group 1 the barycentre showed a deviation to the right (6 ± 2.56 mm) on the latero-lateral axis and anteriorly (20.83 ± 3.76 mm) on the anterior-posterior axis. In the rehabilitation group (Group 2) the barycentre showed a deviation to the right (5.08 ± 2.06 mm) on the latero-lateral axis and anteriorly (13.17 ± 3.61 mm) on the anterior-posterior axis. The control group showed minor displacement of the barycentre on both axes (0.54 ± 0.44 mm on latero-lateral axis and 0.44 ± 0.37 mm on the anterior-posterior axis). The deviations were recorded taking into account the ideal barycentre for each subject.

## Discussion

Our study aims to investigate the static plantar pressure and functional capacity in children who had suffered unilateral femoral shaft fractures and required surgery (by using TEN implants). The assessments were performed 1 month after implant removal. We had two groups of patients. One group was represented by patients who did not attend inpatient rehabilitation after initial surgery. Although the orthopaedic surgeon recommended rehabilitation, the children did not take part in this programme. That was due to the lack of parents’ and adolescents’ compliance to medical recommendations, or because the patients lived far away from a rehabilitation centre. The other study group (rehabilitation group) consisted in children who have followed an inpatient physical exercise programme after the initial surgery. We hypothesized that there would be differences between the children with operatively treated unilateral femoral shaft fractures and healthy controls regarding the static plantar pressure parameters and 6MWT, with better outcomes for the rehabilitation group.

Seven months after surgery the patients in both groups had still an unequal weight load distribution on the feet with a significantly lower weight load on the affected limb. In patients who did not attend rehabilitation, when considering the affected limb, we noticed an increased weight load on the fifth metatarsal head with a significantly decreased weight load on the heel and first metatarsal head. In contrast, in rehabilitation group, the foot loading distribution begins to rebalance; smaller differences from the normal ranges were noticed for the heel and the first metatarsal head.

When compared to healthy controls pronounced changes in weight load distribution were also recorded on the non-affected foot. The patients had a higher weight load on the fifth metatarsal head and lower weight load on the heel and first metatarsal head. These differences are also more pronounced in the group without rehabilitation.

We noticed no significant differences between the loading on the non-affected foot between rehabilitation group and controls. That fact can be due to the balancing of load distribution and a lower overuse of the right limb. After the rehabilitation programme Group 2 patients started to regain the strength of the affected limb and a more accurate balance control.

In literature there are only few studies that assess the distribution of plantar pressure in patients that had suffered a lower extremity fracture. The study of Jaarsma et al. [25] investigated the compensatory gait of adult patients with a femoral rotational malalignment caused by unilateral femoral shaft fractures stabilised internally by means of intramedullary nailing. Our study had in view paediatric patients with unilateral femoral shaft fractures treated by TEN implants. A recent study of Mehlhorn et al. (2017) analysing the plantar pressure after Lisfranc fracture-dislocation in adult patients suggests that a greater attention should be attributed during rehabilitation in order to restore the affected lower leg strength [26]. Our research sustains the necessity of a rehabilitation programme in the management of children who had suffered unilateral femoral shaft fractures, even though they were treated by a mini-invasive surgical technique.

In the current study we also analysed the deviation of the barycentre on the anterior-posterior and latero-lateral axes for the patients. Children who had suffered a left femoral shaft fracture had an increased deviation to the right and anteriorly. This suggests a higher support on the anterior part of the non-affected limb. The deviation had lower values in patients who attended rehabilitation. The plantar pressure distribution on each foot showed a higher loading on non-affected limb compared with the affected one in both patients groups, with a smaller difference in the rehabilitation group.

In the same time our study had in view the assessment of functional capacity by using the 6MWT. In comparison to healthy children, the 6MWT was significantly lower in paediatric patients with femoral shaft fractures even 7 months after surgery. Although patients who followed rehabilitation had an improved functional capacity, the values were still decreased when compared to controls. The study of Kwasnicki et al. (2015) examining the functional capacity in adult patients with open tibial fractures revealed that walking quality continues to improve 12 months postoperatively [27].

After the analysis of study results we recommend that the patients should continue to perform an exercise programme in order to restore the normal plantar distribution, to improve their postural stability and to regain the optimal physical performance. The children with surgically treated femoral fractures should perform a physical exercise programme including an inpatient rehabilitation phase and continued as a long-term home programme. The rehabilitation recommendations focus on regaining balance and a proper symmetric plantar pressure. The aim of physical exercise programme was to decrease the load on the fifth metatarsal, to increase the load on heel and to gain a better lateral stability. We recommended strengthening exercises for calf muscles and tibialis anterior in order to balance the muscle tone. For lateral stability in standing, exercises for strengthening fascia lata, iliotibial tract and tibialis anterior are extremely useful.

Non-adherence to recommended therapy (in this case to the physical exercise programme) represents a factor that limited the optimal functional outcomes. Adherence can thus reduce the efficacy of therapy interventions and functional outcomes regardless of pathology [28, 29]. Although the small number of patients enrolled in our study might be considered as a limitation, the selective inclusion and exclusion criteria allowed us to avoid biases and to obtain a more accurate analysis of the plantar pressure and functional capacity. Another limitation of our study is represented by the lack of gait analysis and dynamic plantar pressure assessment.

## Conclusions

When compared to healthy controls children with surgically treated unilateral femoral shaft fractures, although assessed after 7 months, had a different weight distribution on the feet and a decreased functional capacity. However, these differences are smaller in the group that have also performed rehabilitation. An exercise-based programme included in the management of children with femoral shaft fractures is important for regaining their functional level. We have in view a future study assessing these patients at least 1 year after the fracture.

## Supplementary information

**Additional file 1.** Measurement procedure of plantar pressure.

**Additional file 2.** Raw data.

## Data Availability

All data generated or analysed during this study are included in this published article [and its additional files].

## Abbreviations

6MWT: 6-min walk test
BMI: Body mass index
MT1: First metatarsal head
MT5: Fifth metatarsal head
NS: Not significant
TEN: Titanium elastic nailing
