# Supplementary figures and images for: Static plantar pressure and functional capacity in children with femoral shaft fractures treated by titanium elastic nailing
Source: BMC Musculoskelet Disord. 2019 Nov 26;20:565. doi: 10.1186/s12891-019-2951-z (PMC6880557; doi:10.1186/s12891-019-2951-z)

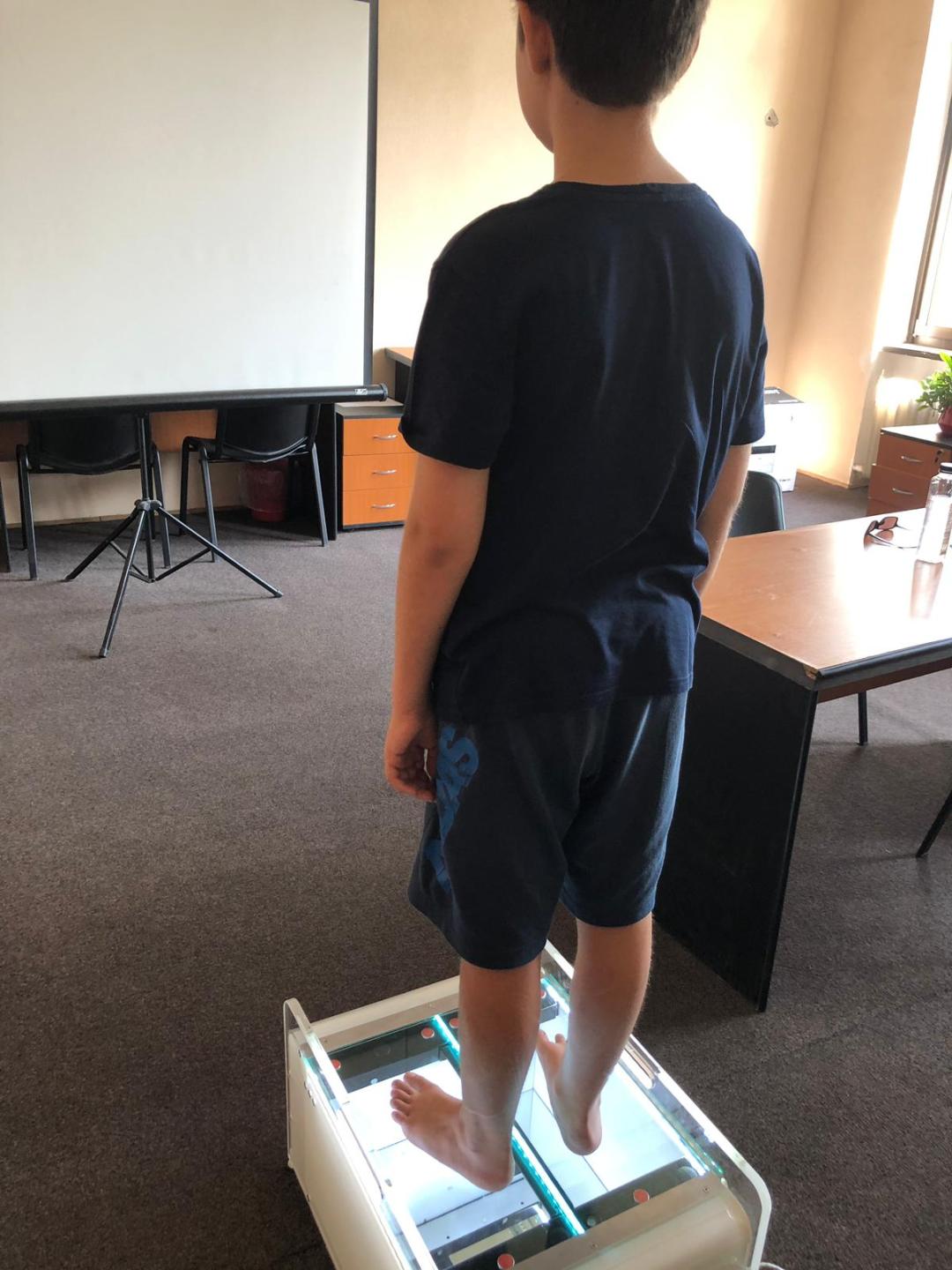

Supplement: Supplementary file 1 — Additional file 1. Measurement procedure of plantar pressure. [file 12891_2019_2951_MOESM1_ESM.jpg]
