# Supplementary material for: Static plantar pressure and functional capacity in children with femoral shaft fractures treated by titanium elastic nailing
Source: BMC Musculoskelet Disord. 2019 Nov 26;20:565. doi: 10.1186/s12891-019-2951-z (PMC6880557; doi:10.1186/s12891-019-2951-z)
Supplement: Supplementary file 2 — Additional file 2. Raw data. [file 12891_2019_2951_MOESM2_ESM.docx]

Raw data

| ID | Group | Gender | Age | Height  cm | Weight  (kg) | BMI  (kg/m^2^) | Site fracture | Leg length difference (cm) | R_foot_load (%) | R_MT1 (%) | R_MT5 (%) | R_Heel (%) | L_foot_load (%) | L_MT1 (%) | L_MT5 (%) | L_Heel (%) | Bary_x_R | Bary_x_F | MWT (m) |
| --- | --- | --- | --- | --- | --- | --- | --- | --- | --- | --- | --- | --- | --- | --- | --- | --- | --- | --- | --- |
| B L | 1 | 1 | 12 | 148 | 38 | 17,35 | Left | 1,1 | 51 | 23 | 36 | 41 | 49 | 31 | 44 | 25 | 5 | 24 | 402 |
| F V | 1 | 1 | 16 | 173 | 69 | 23,05 | Left | 0,9 | 53 | 24 | 42 | 34 | 47 | 21 | 58 | 21 | 2 | 21 | 457 |
| EE | 1 | 1 | 14 | 153 | 49 | 20,93 | Left | 0,4 | 55 | 22 | 44 | 36 | 45 | 23 | 54 | 23 | 4 | 19 | 389 |
| AC | 1 | 2 | 15 | 165 | 54 | 19,83 | Left | 0,7 | 56 | 21 | 37 | 42 | 44 | 23 | 47 | 20 | 5 | 27 | 377 |
| BD | 1 | 1 | 17 | 182 | 65 | 19,62 | Left | 1 | 59 | 19 | 38 | 43 | 41 | 23 | 58 | 19 | 6 | 14 | 466 |
| BV | 1 | 1 | 17 | 179 | 68 | 21,22 | Left | 0,5 | 61 | 21 | 40 | 39 | 39 | 27 | 46 | 27 | 8 | 20 | 421 |
| MM | 1 | 1 | 11 | 150 | 41 | 18,22 | Left | 1,2 | 54 | 24 | 36 | 40 | 46 | 27 | 48 | 25 | 6 | 25 | 390 |
| VI | 1 | 1 | 16 | 176 | 67 | 21,63 | Left | 0,9 | 55 | 18 | 42 | 38 | 45 | 21 | 51 | 28 | 2 | 23 | 489 |
| TV | 1 | 1 | 12 | 144 | 40 | 19,29 | Left | 0,8 | 57 | 22 | 44 | 36 | 43 | 25 | 52 | 23 | 8 | 21 | 356 |
| BI | 1 | 2 | 13 | 140 | 42 | 21,43 | Left | 0,7 | 60 | 20 | 37 | 43 | 40 | 22 | 57 | 21 | 10 | 21 | 345 |
| CS | 1 | 2 | 16 | 169 | 56 | 19,61 | Left | 0,6 | 56 | 19 | 34 | 47 | 44 | 24 | 55 | 21 | 9 | 20 | 407 |
| FL | 1 | 1 | 17 | 180 | 67 | 20,68 | Left | 0,7 | 58 | 18 | 38 | 44 | 42 | 21 | 57 | 22 | 7 | 15 | 451 |
| MD | 2 | 1 | 12 | 150 | 41 | 18,22 |  | 1 | 52 | 34 | 16 | 50 | 48 | 36 | 14 | 50 | 1 | 0,1 | 453 |
| AA | 2 | 1 | 17 | 173 | 68 | 22,72 |  | 0,5 | 53 | 32 | 17 | 51 | 47 | 31 | 16 | 53 | 1 | 0,2 | 678 |
| TS | 2 | 1 | 13 | 152 | 49 | 21,21 |  | 1,2 | 49 | 37 | 14 | 49 | 51 | 35 | 16 | 49 | 0 | 0,6 | 498 |
| TT | 2 | 2 | 15 | 167 | 51 | 18,29 |  | 0,9 | 52 | 34 | 17 | 47 | 48 | 36 | 17 | 47 | 0,5 | 1 | 401 |
| AB | 2 | 1 | 17 | 182 | 70 | 21,13 |  | 0,8 | 55 | 35 | 15 | 50 | 45 | 34 | 16 | 50 | 1 | 0,2 | 577 |
| OV | 2 | 1 | 17 | 180 | 74 | 22,84 |  | 0,7 | 45 | 33 | 18 | 49 | 55 | 35 | 14 | 51 | 0 | 0,1 | 551 |
| IE | 2 | 1 | 14 | 149 | 40 | 18,02 |  | 0,6 | 54 | 29 | 18 | 53 | 46 | 28 | 19 | 53 | 1 | 0,6 | 450 |
| LD | 2 | 1 | 16 | 176 | 65 | 20,98 |  | 0,7 | 55 | 30 | 17 | 53 | 45 | 36 | 16 | 48 | 1 | 1,2 | 581 |
| AI | 2 | 1 | 12 | 146 | 39 | 18,30 |  | 1,5 | 50 | 35 | 17 | 48 | 50 | 29 | 21 | 54 | 0,5 | 0,1 | 405 |
| DD | 2 | 2 | 11 | 141 | 45 | 22,63 |  | 0,5 | 51 | 32 | 17 | 51 | 49 | 30 | 19 | 51 | 0,4 | 0,5 | 383 |
| VS | 2 | 2 | 16 | 168 | 57 | 20,20 |  | 0,4 | 47 | 29 | 21 | 54 | 53 | 31 | 17 | 52 | 0,1 | 0,6 | 479 |
| EA | 2 | 1 | 16 | 180 | 69 | 21,30 |  | 0,7 | 49 | 32 | 19 | 49 | 51 | 32 | 19 | 49 | 0 | 0,1 | 610 |
| CR | 3 | 2 | 15 | 164 | 50 | 18,59 | Left | 1,1 | 55 | 25 | 35 | 43 | 45 | 38 | 30 | 32 | 6 | 21 | 390 |
| SD | 3 | 2 | 16 | 170 | 57 | 19,72 | Left | 0,7 | 52 | 22 | 27 | 51 | 48 | 26 | 34 | 40 | 9 | 12 | 415 |
| FR | 3 | 1 | 12 | 149 | 41 | 18,47 | Left | 0,8 | 51 | 30 | 22 | 48 | 49 | 32 | 27 | 41 | 5 | 15 | 433 |
| AS | 3 | 1 | 16 | 174 | 67 | 22,13 | Left | 0,8 | 52 | 32 | 22 | 46 | 42 | 31 | 26 | 43 | 3 | 17 | 498 |
| FR | 3 | 1 | 16 | 177 | 66 | 21,07 | Left | 0,5 | 53 | 31 | 20 | 49 | 47 | 30 | 29 | 41 | 2 | 11 | 485 |
| GT | 3 | 1 | 16 | 181 | 72 | 21,98 | Left | 0,7 | 55 | 29 | 23 | 48 | 45 | 25 | 45 | 30 | 6 | 8 | 501 |
| HE | 3 | 1 | 12 | 150 | 40 | 17,78 | Left | 1,7 | 51 | 32 | 21 | 47 | 49 | 32 | 30 | 38 | 5 | 12 | 432 |
| BN | 3 | 2 | 13 | 141 | 43 | 21,63 | Left | 1,4 | 58 | 26 | 25 | 49 | 42 | 29 | 34 | 37 | 7 | 15 | 369 |
| ED | 3 | 1 | 17 | 183 | 68 | 20,31 | Left | 0,9 | 54 | 33 | 20 | 47 | 46 | 30 | 31 | 39 | 2 | 9 | 410 |
| AR | 3 | 1 | 17 | 180 | 67 | 20,68 | Left | 1,2 | 58 | 27 | 29 | 44 | 42 | 27 | 35 | 38 | 6 | 12 | 465 |
| MO | 3 | 1 | 14 | 151 | 44 | 19,30 | Left | 0,7 | 54 | 28 | 30 | 42 | 46 | 24 | 38 | 38 | 4 | 11 | 447 |
| SF | 3 | 1 | 11 | 143 | 43 | 21,03 | Left | 1,2 | 52 | 30 | 27 | 43 | 48 | 29 | 36 | 35 | 6 | 15 | 425 |
